# Cavernous Tumor of the Orbit, Complicated with a Large Sanguineous Cyst; Successful Removal without Injury to the Globe or Optic Nerve

**Published:** 1871-01

**Authors:** E. L. Holmes

**Affiliations:** Professor Ophthalmology in Rush Medical College; Surgeon to the Chicago Charitable Eye and Ear Infirmary


### Chicago Medical Journal.

A MONTHLY RECORD OF

*Medicine, Surgery, and the Collateral Sciences.*

EDITED BY J. ADAMS ALLEN, M.D., LL.D.; AND WALTER HAY, M.D.

VOL. XXVIII.—JANUARY, 1871.—No. 1.

### Original Communications.

ARTICLE I.—*Cavernous Tumor of the Orbit, Complicated with a large Sanguineous Cyst. Successful Removal without injury to the Globe or the Optic Nerve.* By E. L. HOLMES, M.D., Professor Ophthalmology in Rush Medical College; Surgeon to the Chicago Charitable Eye and Ear Infirmary.

Firm and well defined tumors, having an internal structure almost identical with that of the cavernous portion of the penis, are quite rarely found in any portion of the body. But five cases, I think, of such tumors in the orbit are on record.

The following case must be considered as remarkable for its great rarity and for its singular complication with a large sanguineous cyst.

Mrs. B., æt. 48, mother of five children, consulted me in June last with the following objective symptoms. The left eye—the right being perfectly normal—protruded from the orbit to such an extent that on opening the lids widely, the patient was unable to close them without manual assistance. The cornea, normal in appearance, was turned extensively inward and slightly downward. The pupil was moderately dilated and immovable; the conjunctiva but little congested and not œdematous; the upper lid much distended; the lower one somewhat thickened. There was scarcely any motion of the globe.

At the outer and upper portion of the orbit there was a large tumor projecting between the globe and orbital wall. This tumor, from its position, and great movability, could be recognized almost certainly as the lachrymal gland, pushed forward by some growth behind.

The ophthalmoscope revealed numerous radiating lines in the lens, which materially impaired vision and rendered a view of the fundus indistinct. The enormous exophthalmos had slowly developed during the past year and a half.

The patient had never suffered actual pain, although there was a disagreeable sense of distention and weight.

The cause of the disease was absolutely unknown. There was no hereditary tendency to cancerous disease. The patient's general health had been excellent, until she had been informed some months since, by a physician, that the disease was cancerous. This had depressed her spirits, broken her rest, and destroyed in a great measure her appetite.

She was consequently in an enfeebled condition on coming under my care.

The diagnosis was doubtful. In consultation with Prof. M. Gunn, M.D., and W. C. Hunt, M.D., to whom I am greatly indebted for advice and assistance, it was decided to make an incision over the tumor and examine the microscopic structure of the tissue beneath.

On making the incision between the lid and globe at the upper and outer portion of the orbit, the lachrymal gland at once protruded almost entirely through the wound in the conjunctiva. The gland was extirpated to facilitate further steps. On passing the finger less than half an inch through the wound thus made, I discovered a well defined and quite hard tumor nearly filling the outer half of the orbit in front, and extending to an unknown distance posteriorly. Dr. Hunt made a microscopical examination of the tissue around this tumor, but discovered no cells of a malignant character. I therefore made an incision into the tumor, when, at least two and a half drachms of bloody fluid escaped. By means of a pair of forceps and the handle of the scalpel the entire cyst, although totally collapsed, was readily removed.

On again passing the finger into the wound I discovered another tumor situated quite deep at the lower and outer portion of the orbit. During and after the removal of the cyst the optic nerve could be felt with the tip of the finger for a distance of at least half an inch.

The tumor was removed from its deep position with the greatest facility without dissection, almost as if it had been a loose foreign body in the orbit. The globe receded at once to its natural position.

It must certainly be regarded as remarkable, not only that during the whole operation a very small quantity of blood was lost, but that the tumor seemed to have scarcely any attachment with the adjoining tissues.

Neither the optic nerve nor muscles were injured during the operation.

At the end of two weeks the wound had nearly healed; the motions of the lids and globe, although the orbicularis and recti muscles had been enormously stretched, had become quite natural. I subsequently heard that the motions of the eye were almost absolutely normal.

The tumor was one and a quarter inches long, two-thirds of an inch wide, and three-eighths of an inch thick, resembling in form and hardness a testis. It was surrounded with a very thin capsule. On making a small incision into it, I found it divided into minute cavities, filled with blood and separated by very thin walls. The connections between these spaces were not free, since pressure of the tumor forced the blood from the divided cavities alone. Deeper incisions and pressure caused more blood to escape.

The microscopic appearances, as examined by Dr. Hunt, were almost identical with those of the corpus cavernosum of the penis, although there seemed to be no true veins, arteries, nor nerves. The inclosing membrane or capsule, although thin, was very richly supplied with vessels; there was difficulty in following the entrance of these vessels into the tumor. No portion of the tumor, showing connection with the cyst, could be discovered.

